# Functional Analysis of Esterase *TCE2* Gene from *Tetranychus cinnabarinus* (Boisduval) involved in Acaricide Resistance

**DOI:** 10.1038/srep18646

**Published:** 2016-01-04

**Authors:** Li Shi, Peng Wei, Xiangzun Wang, Guangmao Shen, Jiao Zhang, Wei Xiao, Zhifeng Xu, Qiang Xu, Lin He

**Affiliations:** 1 Key Laboratory of Entomology and Pest Control Engineering, College of Plant Protection, Southwest University, Chongqing, China; 2 Department of Biology, Abilene Christian University, Abilene, Texas, U.S.A.

## Abstract

The carmine spider mite, *Tetranychus cinnabarinus* is an important pest of crops and vegetables worldwide, and it has the ability to develop resistance against acaricides rapidly. Our previous study identified an esterase gene (designated *TCE2*) over-expressed in resistant mites. To investigate this gene’s function in resistance, the expression levels of *TCE2* in susceptible, abamectin-, fenpropathrin-, and cyflumetofen-resistant strains were knocked down (65.02%, 63.14%, 57.82%, and 63.99%, respectively) via RNA interference. The bioassay data showed that the resistant levels to three acaricides were significantly decreased after the down-regulation of *TCE2*, indicating a correlation between the expression of *TCE2* and the acaricide-resistance in *T. cinnabarinus*. *TCE2* gene was then re-engineered for heterologous expression in *Escherichia coli*. The recombinant TCE2 exhibited α-naphthyl acetate activity (483.3 ± 71.8 nmol/mg pro. min^−1^), and the activity of this enzyme could be inhibited by abamectin, fenpropathrin, and cyflumetofen, respectively. HPLC and GC results showed that 10 μg of the recombinant TCE2 could effectively decompose 21.23% fenpropathrin and 49.70% cyflumetofen within 2 hours. This is the first report of a successful heterologous expression of an esterase gene from mites. This study provides direct evidence that *TCE2* is a functional gene involved in acaricide resistance in *T. cinnabarinus.*

The carmine spider mite, *Tetranychus cinnabarinus* (Boisduval), is one of the most damaging pest mites in agriculture and forestry. It distributes worldwide, and feeds on more than 100 crops or plants grown in the field or greenhouse, such as beans, aubergines, peppers, tomatoes, and cucurbits^1^,^2^. Because of its morphological, biological, and molecular characteristics are quite similar to those of the two-spotted mite, *Tetranychus urticae*, therefore, some researchers also considered them as two forms (red and green) of a single species (*T. urticae*)^3^,^4^. For many years, the control of this mite has traditionally relied on sprays of acaricides. But *T. cinnabarinus* can rapidly develop resistance against acaricides after only a few generations^5^.

Carboxylesterases (CarEs) constitute a class of enzymes that hydrolyze chemicals containing such functional groups as carboxylic acid ester, amide and thioester^6^, which are widely distributed in microbes, plants and animals. These enzymes hydrolyze chemicals containing carboxylic esters to the corresponding component alcohols and acids^6^. In insects, CarEs have diverse biological functions, such as metabolism of specific hormones and detoxification of dietary and environmental xenobiotics^7^. Many studies have reported that the elevation of esterase activity through gene amplification or up-regulated transcription accounts for some degree of resistance against insecticides in insects^8^. Elevation of esterase activity through up-regulated esterase transcription and point mutations within esterase genes are two known mechanisms of esterase-mediated insecticide resistance^9^,^10^. The synergistic action of several CarEs in conferring resistance against acaricides is known in some mites and ticks. For instance, it has been certified that CarEs contribute to resistance to bifenthrin, fenpyroximate, and spirodiclofen in *Tetranychus urticae*^11^,^12^,^13^ and phoxim in *Panonychus citri*^14^. Significantly higher activity of esterases has been observed in resistant populations of *Rhipicephalus bursa*^15^. Esterases are involved in pyrethroid and organophosphate resistance in *Riphicephallus microplus*^16^. In many cases, gene amplification following an exposure to xenobiotic compounds enhances expression levels of metabolic enzymes that, in turn, trigger resistance^17^,^18^.

In recent years, RNA interference (RNAi) has been identified as a universal gene-silencing mechanism in insects^19^,^20^. RNAi-based technology has shown great potential in controlling insect pests by silencing vital genes^21^,^22^. RNAi has been successfully used to investigate the function of esterases, P450s, and GSTs in some insects, such as *Aphis gossypii*^23^, *Sitobion avenae*^24^, *Helicoverpa armigera*^22^, and *Bemisia tabaci*^25^. However, little has been done to study RNAi-based technology in mites. In *T. urticae*, the effects of RNAi of the distal-less gene, which is involved in appendage specification, were firstly investigated by delivering dsRNA via microinjection^26^; and several lethal genes were screened by the systemic delivery of dsRNA via leaf disc feeding^27^.

Heterologous expression was also used to investigate the function of some detoxification genes in insects and mites. For instance, two cytochrome P450 genes *CYP392E10*^28^ and *CYP392A16*^29^ in *T. urticae*, which were functionally expressed in *Escherichia coli*, could metabolize spirodiclofen, spiromesifen, and abamectin. *CYP6G1* from *Drosophila melanogaster* is capable of metabolizing both DDT (organochlorine) and imidacloprid (neonicotinoid)^30^. Carboxylesterase E4 (*CbE E4*) of the peach-potato aphid, *Myzus persicae* was successfully expressed in *E. coli*, and the recombinant enzyme hydrolyzed carbaryl by 64% within 2.5 h and malathion by 80% within 1.25 h^31^. *D1CarE5* of thermophilic bacterium, *Alicyclobacillus tengchongensis*, hydrolyzed 5 mg malathion/L by 50% within 25 min and by 89% within 100 min^32^.

In our previous studies, Sun *et al.*^33^ identified and cloned a novel esterase gene *TCE2* from *T. cinnabarinus*, and this particular gene was over-expressed in the adults of abamectin-, fenpropathrin-, and omethoate-resistant *T. cinnabarinus* strains compared to susceptible strain^34^. More interestingly, this *TCE2* gene was inducible with the applications of these three acaricides^34^. These findings strongly suggested the potential involvement of *TCE2* gene in acaricide-resistance in *T. cinnabarinus*. To further investigate the function of *TCE2* involved in the formation of acaricide resistance of *T. cinnabarinus*, in this study, we successfully conducted RNA interference (RNAi) of this gene through dsRNA feeding. Bioassay results showed that after down-regulation of this gene, the susceptibilities of the mites against acaricides were increased, indicating a correlation between the function of *TCE2* and the tolerance to acaricides of mites. Then the *TCE2* gene was expressed in *Escherichia coli*. The activity of this heterologously expressed enzyme could be inhibited by acaricides; and the results from GC- and HPLC-analysis indicated that the recombinant TCE2 protein could effectively degrade fenpropathrin and cyflumetofen. Our study, for the first time, successfully expressed a functional esterase gene of mites. The results of this study provided direct evidence that *TCE2* is involved in acaricide resistance and cross-resistance in *T. cinnabarinus*.

## Results

### *TCE2*-dsRNA knockdown efficiency in susceptible and three resistant strains

**Figure 1:**
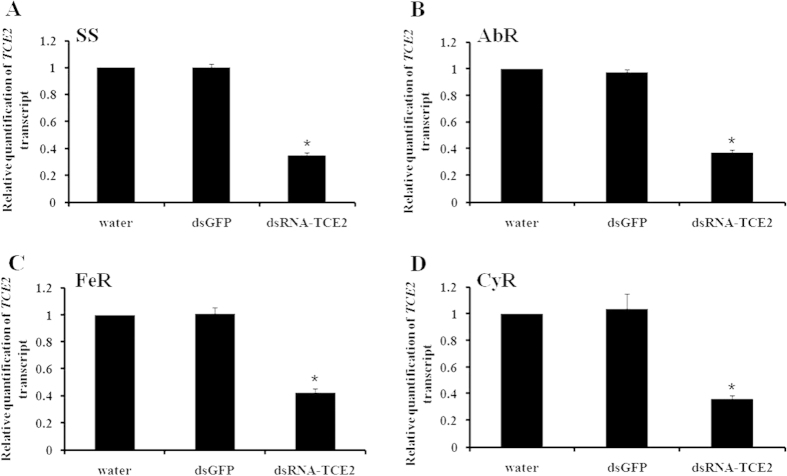
The dsRNA-mediated suppression of *TCE2* transcript expression in susceptible and three resistant *T. cinnabarinus* strains. (**A**) susceptible strain (SS), (**B**) abamectin-resistant strain (AbR), (**C**) fenpropathrin-resistant strain (FeR), (**D**) cyflumetofen-resistant strain (CyR). The final concentration of dsRNA was 1000 ng/μL. Error bars represent the standard error of the calculated mean based on three biological replicates. ^*^indicates significant differences compared to water as determined by independent-sample t-test (*P* < 0.05).

After feeding of dsRNA-*TCE2*, the mRNA relative expression levels were detected by using qRT-PCR to investigate the knockdown efficiency of the *TCE2* gene’s expression in susceptible and three resistant strains. The results showed that the transcript levels of *TCE2* gene in susceptible, abamectin-, fenpropathrin-, and cyflumetofen-resistant strains were significant decreased 65.02%, 63.14%, 57.82% and 63.99%, respectively, compared with the controls (water and dsGFP) (Fig. 1). These results demonstrated that the *TCE2* transcripts were successfully knocked down with RNAi in *T. cinnabarinus*.

### Susceptibility test of *T. cinnabarinus* to acaricides after RNAi of *TCE2*

**Figure 2:**
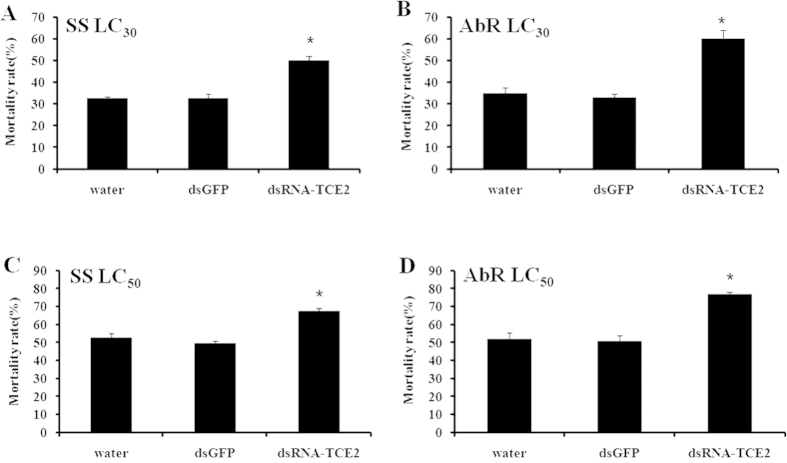
Effect of *TCE2* gene knockdown on mortality of susceptible strain (SS) and abamectin-resistant strain (AbR). (**A**,**C**) the mortality of SS exposed to abamectin with concentrations of LC_30_ and LC_50_; (**B**,**D**) the mortality of AbR exposed to abamectin with concentrations of LC_30_ and LC_50_. The final concentration of dsRNA was 1000 ng/μL. Error bars represent the standard error of the calculated mean based on three biological replicates. ^*^indicates significant differences compared to water as determined by independent-sample *t*-test (*P* < 0.05).

**Figure 3:**
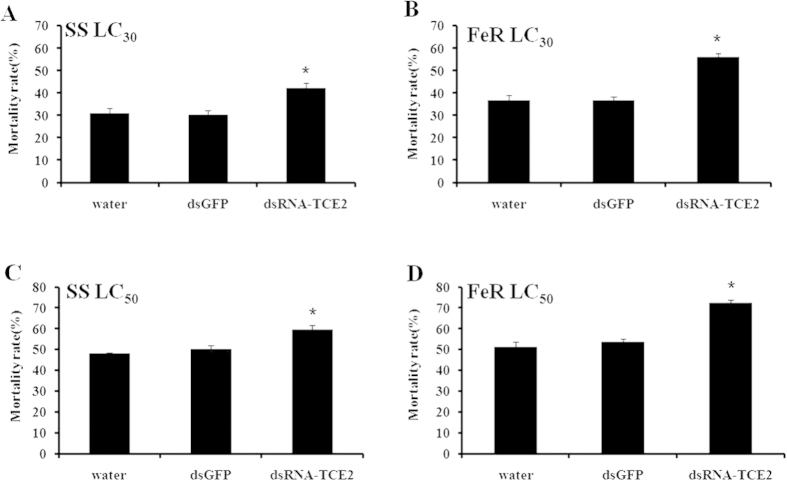
Effect of *TCE2* gene knockdown on mortality of susceptible strain (SS) and fenpropathrin-resistant strain (FeR). (**A**,**C**) the mortality of SS exposed to fenpropathrin with concentrations of LC_30_ and LC_50_; (**B**,**D**) the mortality of FeR exposed to fenpropathrin with concentrations of LC_30_ and LC_50_. The final concentration of dsRNA was 1000 ng/μL. Error bars represent the standard error of the calculated mean based on three biological replicates. ^*^indicates significant differences compared to water as determined by independent-sample *t*-test (*P* < 0.05).

**Figure 4:**
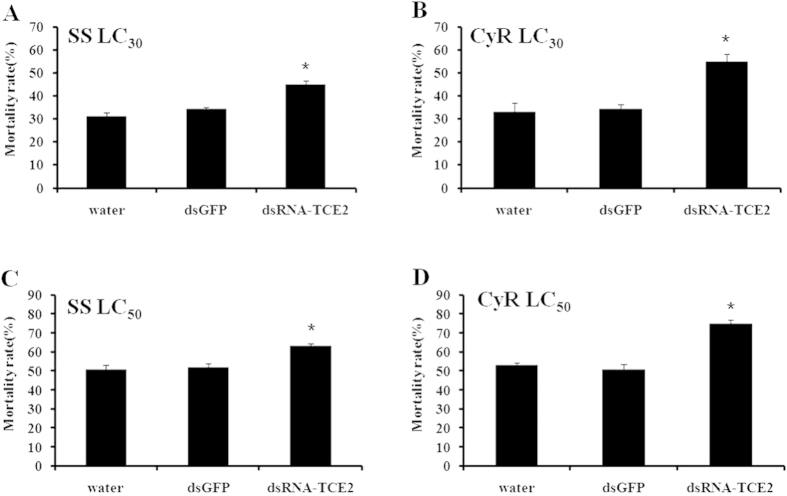
Effect of *TCE2* gene knockdown on mortality of susceptible strain (SS) and cyflumetofen-resistant strain (CyR). (**A**,**C**) the mortality of SS exposed to cyflumetofen with concentrations of LC_30_ and LC_50_; (**B**,**D**) the mortality of CyR exposed to cyflumetofen with concentrations of LC_30_ and LC_50_. The final concentration of dsRNA was 1000 ng/μL. Error bars represent the standard error of the calculated mean based on three biological replicates. ^*^indicates significant differences compared to water as determined by independent-sample *t*-test (*P* < 0.05).

The susceptibilities to three acaricides after 48 h post-feeding of dsRNA-*TCE2* were detected by RCV method. When treated with LC_30_ and LC_50_ of abamectin, the mortality increased from 32.49% and 52.58% to 50.09% and 67.52%, respectively, in susceptible mites; while the mortality increased significantly from 34.71% and 52.0% to 59.75% and 76.82%, respectively, in the abamectin-resistant strain (Fig. 2). When the concentrations of LC_30_ and LC_50_ of fenpropathrin were applied to susceptible and fenpropathrin-resistant strains, the mortality increased from 30.98% and 48.12% to 42.25% and 59.56%, respectively, in the susceptible strain; whereas the death rate increased dramatically from 36.86% and 51.28% to 55.95% and 72.45%, respectively, among fenpropathrin-resistant mites (Fig. 3). When treated with LC_30_ and LC_50_ of cyflumetofen, the mortality increased from 30.99% and 50.79% to 44.84% and 63.36%, respectively, in the susceptible strain; while the mortality increased significantly from 33.05% and 53.14% to 54.73% and 74.78%, respectively, in cyflumetofen-resistant strain (Fig. 4). There was no significant mortality difference between two controls (water and dsGFP) in susceptible and three resistant strains (Figs 2,3,4). It is worth pointing out that, after treated with dsRNA-*TCE2*, the increased mortalities in three resistant strains were all higher than that in susceptible strain, indicating that the resistant mites were more sensitive to acaricides when the *TCE2* was down-regulated. These results revealed that RNAi of *TCE2* gene had an obvious effect on the mortality of *T. cinnabarinus* to acaricides, that is, increasing the susceptibilities of mites to all three acaricides.

### Expression and purification of recombinant *TCE2*

**Figure 5:**
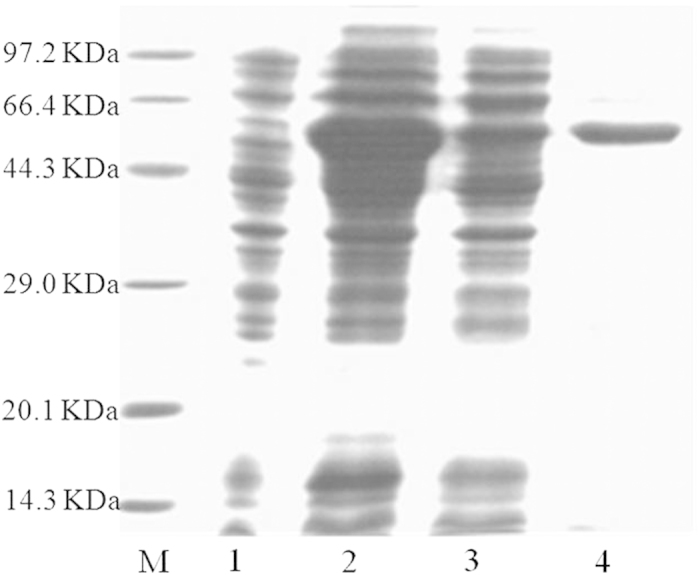
SDS-PAGE analysis of recombinant TCE2 expression in *E. coli* (DE3). Lane M: low-molecular weight markers; Lane 1: recombinant TCE2 without IPTG induction; Lane 2: recombinant TCE2 with IPTG induction; Lane 3: soluble protein; Lane 4: purified by Ni^2 + ^-NTA.

In order to functionally express *TCE2* gene in *E. coli*, the signal peptide sequence of *TCE2* was removed for the recombinant protein expression. A high level expression was observed in BL21(DE3) cells transformed with pCold II-*TCE2* with IPTG induction when cultured at 15 °C for 24 h, and after gentle sonication on ice, the recombinant TCE2 was released into the supernatant. A new specific band about 63 kDa was observed in the SDS-PAGE analysis when the whole cells lysate was applied for the experiment (Fig. 5). Recombinant TCE2 purification was performed by Ni^2+^-NTA agarose gel column at 4 °C. The SDS-PAGE analysis showed that the recombinant TCE2 was well purified with only one observed band around 63 kDa, which was close to the value for the calculated molecular weight of the deduced amino acid sequence of the TCE2 protein (Fig. 5).

### Esterase-activity assay for recombinant TCE2 and crude enzyme

The esterase activities of recombinant TCE2 and crude enzymes extracted from mites were measured using α-naphthyl acetate (α-NA) as the substrate. The amount of the recombinant TCE2 was estimated to 0.10 mg/ml of induced culture by Bradford method. The specific activity of recombinant TCE2 is 483.3 ± 71.8 nmol/mg pro.min^−1^, and its *Km* was determined to be 30.5 ± 5.9 μM. The specific activities of crude enzymes are 233.8 ± 20.3 and 235.4 ± 1 0.3 nmol/mg pro. min^−1^ for *T.cinnabarinus* and *T. urticae* susceptible mites, respectively (Table 1). The recombinant enzyme showed a much higher activity compared with crude enzymes extracted from two susceptible mite species.

### Inhibition of three acaricides to α-NA degradation mediated by the recombinant TCE2

**Figure 6:**
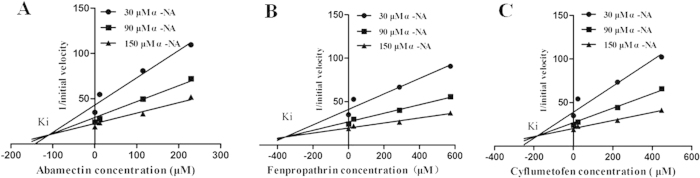
Dixon plot analysis for the inhibition of α-NA conjugating activity of TCE2 by different abamectin, fenpropathrin, and cyflumetofen concentrations. Three different concentrations of α-NA (30, 90 and 150 μM) and four different concentrations of (**A**) abamectin (0, 12, 115, 230 μM), (**B**) fenpropathrin (0, 29, 287, 574 μM), (**C**) cyflumetofen (0, 23, 224, 447 μM) were used and data are mean of three replicates ± S.D. Analysis denoted a competitive type of inhibition and the Ki were determined as (**A**) 109.5 ± 10.45 μM, (**B**) 356.23 ± 35.36 μM, (**C**) 182.45 ± 17.68 μM.

IC_50_s of three acaricides on inhibiting the esterase activity of TCE2 (α-NA as substrates) were tested. All three acaricides showed significant inhibition on α-NA degradation catalyzed by the recombinant TCE2 (Table 2), from which abamectin had the highest inhibiting effect; and its efficiency was as about 2- and 4-times as that of cyflumetofen and fenpropathrin, respectively. Dixon plot analysis with various concentrations of α-NA, abamectin, fenpropathrin, and cyflumetofen were performed to investigate inhibition patterns of these three acaricides. The resulting linear curves converged above the x axis for all three acaricides (Fig. 6), demonstrating that all three inhibitions were competitive. The inhibitor constants (Ki) for abamectin, fenpropathrin and cyflumetofen were calculated as 109.5 ± 10.45 μM, 356.23 ± 35.36 μM and 182.45 ± 17.68 μM, respectively (Fig. 6). Interestingly, the competitive inhibition patterns of three acaricides suggest that abamectin, fenpropathrin and cyflumetofen compete with α-NA for the same enzyme active site of TCE2.

### Assessing the capability of TCE2 to metabolize acaricides

In order to determine the hydrolysis activity of recombinant TCE2 protein, HPLC and GC were used to test the metabolism of abamectin, fenpropathrin, and cyflumetofen *in vitro*. Catalytic activity was initially assessed by measuring substrate depletion. The results showed that the decomposition rate of fenpropathrin was 21.31 ± 2.29% when treated with 10 μg of recombinant TCE2 (Table 3). The decomposition rate of cyflumetofen was 30.23 ± 5.82% when incubated with 5 μg of enzyme, and when the amount of recombinant TCE2 was doubled, the rate of cyflumetofen depletion increased to 49.70 ± 11.45% (Table 3). However, it seemed that the recombinant TCE2 was unreactive against abamectin as there was no apparent substrate depletion observed.

## Discussion

In many arthropod pests, resistance to pesticides has been attributed to an increase in detoxifying enzyme activities^35^. Most pesticides in use today are esters of substituted phosphoric, carbamic or cyclopropanecarboxylic acids, and are consequently subject to degradation by esterases^36^. Esterase-mediated insecticide resistance has been reported in more than 30 pest species^37^. In many cases, insecticide resistance is mediated by mutations or up-regulation of involved genes^38^. In mites, Tsagkarakou *et al.*^39^ identified a point mutation (F1538I) in segment 6 of domain III from sodium channel gene, which is known to confer strong resistance to pyrethroids in *T. urticae*. In our previous study, Feng *et al.*^40^ has also reported the same mutation (F1538I) from fenpropathrin-resistant strain of *T. cinnabarinus*. It has been further proven to confer strong resistance against fenpropathrin and its mutation frequency was closely related to the resistant level^41^. Metabolic resistance against pyrethroids mediated by CarEs or P450s is also well documented^42^. In *T. urticae*, mixed function oxidase (MFO) and esterase were confirmed as metabolic factors related to abamectin resistance^43^; and a higher esterase activity was documented in resistance strains against bifenthrin^13^,^44^. Our previous research has shown that the resistance of *T. cinnabarinus* to abamectin and fenpropathrin correlates to an enhanced activity of esterases^45^, but direct proof is still needed to reveal the function of esterase genes in *T. cinnabarinus*, such as *TCE2* gene.

In this study, we took the advantage of RNAi technique to evaluate possible roles of *TCE2* in acaricide-resistance of *T. cinnabarinus*. The dosages of LC_30_ and LC_50_ for susceptible and each resistant strain were used to detect the effect of RNAi on the change of susceptibility of *T. cinnabarinus*. The results showed that a range from 57.82% to 65.02% of the mRNA-expressions of *TCE2* gene were successfully inhibited by feeding dsRNA of *TCE2* to the mites in susceptible and three resistant strains. The mortalities of dsRNA-*TCE2*-fed *T. cinnabarinus* from susceptible and three resistant strains were all increased when exposed to three acaricides, revealing that knocking down *TCE2* gene’s expression decreased the detoxification effect of esterase encoded by *TCE2* on acaricides. It was also worth taking into the consideration that, after RNAi treatment, the increased mortalities of three resistant strains were higher than that in susceptible strain, indicating that the resistant mites were more sensitive to acaricides when the *TCE2* gene expression was down-regulated, from which one quite possible reason was that *TCE2* played a crucial role in the development of pesticide resistance in *T. cinnabarinus*. The current result form RNAi is consistent with our previous report that over-expression of *TCE2* gene was associated with acaricide-resistance in mites^34^.

Post-transcriptional gene silencing mediated by RNA interference (RNAi) is a very useful tool to detect the functions of individual gene. Microinjection and oral delivery of dsRNA have been reported to be valuable methods for achieving RNAi in insects^23^,^46^. However, *T. cinnabarinus* mites are too small, and it is difficult to exert microinjections without deleteriously affecting survival rates. Therefore, in this study, we developed a new way of leaf-disc feeding containing dsRNA-*TCE2* to knock down the expression of *TCE2* gene. When 500 ng/μL of dsRNA was applied in RNAi experiments, the average silence-efficiency was about 30% (ranged from 26.5% to 31.8% in three resistant strains; data not shown). However, when the concentration of dsRNA was doubled to 1000 ng/μL for feeding, about 62% of the mRNA expressions of *TCE2* gene were successfully inhibited in resistant strains and 65.0% of the *TCE2* mRNA expression was down-regulated in the susceptible strain. Although the silencing effect observed through RNAi in the current study is quite significant, from which about 60% of *TCE2* gene’s expression was inhibited, it was still incomplete, which is similar to the RNAi effect observed from some other insects^47^. This may be due to the fact that the intake of dsRNA is limited as a certain extent of the degradation of dsRNA happened either in the leaf prior to ingestion or in the body of *T. cinnabarinus* after ingestion. Recently, Allen *et al.*^48^ also found that the saliva of *Lygus lineolaris* is capable of degrading dsRNA. All of these are the possible reasons causing incomplete silencing by dsRNA feeding. However, our results demonstrated that the delivery of dsRNA via leaf-disc feeding could reach 65% knockdown efficacy in mites. Therefore, our current procedure can now be considered as a very effective method to achieve RNAi in *T. cinnabarinus* and maintaining the dsRNA soaked in the leaf-discs with a concentration of 1000 ng/μL is essential for a high RNAi efficiency.

Heterologous expression system was also utilized to investigate the function of the *TCE2* gene. For the first time, we were able to express a mite esterase gene in the *E. coli* expression system. The recombinant TCE2 was successfully over-expressed and purified through modifying the N-terminal sequence by removing the signal peptide; and its enzymatic properties were characterized. Highly specific expression culture conditions and constituents were necessary for TCE2 protein expression. Heterologous expression is a common practice, especially for enzyme studies, to acquire specific gene products, providing opportunities to study specific genes’ functions *in vitro*. Recombinant proteins expressed from different genes have huge differences in terms of their enzyme activities when compared with natural crude enzyme extracts obtained from organisms. Vontas *et al.*^49^ reported that the activities of purified recombinant GSTs ranged from 2- to 6-fold higher than those of partially purified natural GSTs from *Nilaparvata lugens* according to different substrates. In *T. urticae*, the activity of a recombinant P450, CYP392A16, had significantly higher (about 275 fold) enzyme activities than the P450s from crude enzymes of mites when 7-ethoxy-4-trifluoromethylcoumarin was used as the substrate^29^. In our current study, the crude enzyme extracts from susceptible *T. cinnabarinus* showed similar esterase activity (Table 1) to those of laboratory susceptible strain (London) of *T. urticae*^29^. The activity of recombinant TCE2 was a little bit more than 2-fold of the activity of the crude enzyme extracts when α-NA was used as the substrate (Table 1). Although we should not rule out the possibility that the enzyme activity of recombinant TCE2 might change when different substrates would be used, the recombinant TCE2 did not have much higher enzyme activity than crude enzymes from mites. Therefore, other expression systems, such as yeasts and baculovirus, might need to be considered for the expression of the *TCE2* gene in future research. However, our results have clearly indicated that the *TCE2* gene was successfully expressed by *E. coli* expression system, and the recombinant protein showed the specific esterase activity.

The links between esterases and resistance to a number of different classes of pesticides have been well documented^50^. However, direct evidence demonstrating the interaction between enzymes and chemical compounds is limited. The present study provides an approach to characterize the interaction between a detoxification enzyme and pesticides. Acaricide inhibition of α-NA degradation mediated by TCE2 offers a complementary method to study the association of ligands with the recombinant TCE2 active site. Abamectin exhibited high-affinity inhibition of α-NA degradation with IC_50_ values in the lower micromolar range, while fenpropathrin and cyflumetofen were identified as low-affinity inhibitors with IC_50_ values greater than 100 μM^51^. Furthermore, Dixon plot analysis revealed that the inhibition patterns of three acaricides to α-NA degradation mediated by recombinant TCE2 were competitive, indicating that abamectin, fenpropathrin, and cyflumetofen competed with α-NA for the same enzyme active site of TCE2. These results suggested that these three acaricides could be the substrates of TCE2 protein.

Biodegradation is an important environment biotechnology for the treatment of organic pollutants. Because these resistant insects can detoxify many kinds of pesticides, the enzymes encoded by resistant genes of the insects may be useful to degrade pesticide pollutants in the environment^31^. One treatment strategy is the use of some key enzymes to break down pesticide residues^32^. What is more, we now can use this model to study the relationship between some key enzymes and pesticides. In this study, recombinant TCE2 could effectively decompose 21.23% fenpropathrin and 49.70% cyflumetofen with 10 μg protein within 2 hours, whereas abamectin could not be degraded. However, it does not mean that TCE2 has nothing to do with abamectin-resistance in *T. cinnabarinus* because RNAi of *TCE2* expression did reduce the resistance in abamectin-resistant strain. One possible reason might be that the mode of action of TCE2 protein for abamectin-resistance is combining rather than decomposing abamectin, at least in the early stage of their interaction, which could also confer resistance via decreasing/delaying the amount of abamectin reaching to the target site in *T. cinnabarinus*. This inference was supported by other documented research^52^,^53^ and our current result, indicating that abamectin exhibited high-affinity combination with recombinant TCE2. Also, the ability of recombinant TCE2 to hydrolyze fenpropathrin and cyflumetofen does not contradict with the results that three acaricides were able to inhibit the enzyme activity of TCE2 on α-NA. On the contrary, the competitive inhibition patterns of three acaricides to TCE2 enzyme activity revealed by Dixon plot analysis indicates that all three acaricides compete with α-NA for the same enzyme active site of TCE2, supporting the idea that three acaricides can be the substrates of TCE2 protein. Similar results documenting that insecticides/acaricides combined to recombinant proteins and inhibited enzyme activities were also identified in recent studies^29^,^51^,^54^. Taken all together, *TCE2* gene is responsible for acaricide-resistance in *T. cinnabarinus*; and TCE2 protein has extensive effect on the detoxification of diverse acaricides.

In conclusion, our current study provides insights about the role of *TCE2* gene on the abamectin-, fenpropathrin-, and cyflumetofen-resistance development in *T. cinnabarinus* by means of RNA interference, heterologous expression, and chromatographic analysis. RNAi of *TCE2* gene expression increased the susceptibility of *T. cinnabarinus* against abamectin, fenpropathrin, and cyflumetofen, suggesting that *TCE2* gene plays a crucial role in acaricide resistance development. Feeding of dsRNA through leaf-disc can be utilized for RNAi in carmine spider mites, which should be expanded to gene functional study in other phyllophagous mites. Our results also showed that fenpropathrin and cyflumetofen could be metabolized by the recombinant TCE2 protein, whereas abamectin might be combined, not necessary be degraded immediately, by the protein. Both these two different modes of action-mediated mechanisms are associated with acaricides-resistance in *T. cinnabarinus.* Our findings enhance the understanding of abamectin-, fenpropathrin-, and cyflumetofen-resistance mechanisms in *T. cinnabarinus*, and shed new lights to design strategic ways to control resistant mites. For instance, *TCE2* gene could be a principle target for new acaricide design and for the development of specific diagnostics to improve pesticide resistance management strategies since the overexpression of *TCE2* gene could cause cross-resistance in these three acaricides. Furthermore, we could also imagine that the commercialized TCE2 enzyme products might be used as detergent to wash fruits and vegetables for removing pesticide residues and to recover the pesticide-polluted soil and water in the future.

## Methods

### Mite strains

Susceptible strain (SS): the laboratory carmine spider mite population was originally collected from the field of Beibei District, Chongqing, China, then, it was transferred to fresh potted cowpea leaves and kept in artificial climate chamber (pesticide free) for more than 15 years. Abamectin-resistant (AbR), fenpropathrin-resistant (FeR) and cyflumetofen-resistant (CyR) strains were generated from the SS strain with the selection of abamectin, fenpropathrin, and cyflumetofen, respectively, in the laboratory. The rearing conditions were as follows: 26 ± 1 °C temperature, 35–55% relative humidity (RH), and a photoperiod of 14:10 h (L:D).

### Reagents

The 95% abamectin and 92% fenpropathrin were ordered from Bangnong Chemical Company (Guangzhou, China). The 97% cyflumetofen was obtained from Hangzhou Fumeite Plant Protective Ltd. (Hangzhou, China); iQ™ SYBR^®^ Green Supermix was acquired from BIO-RAD, Hercules (CA, USA); pCold II expression vector and EcoR I, Xba I were obtained from Takara (Dalian, China); pGEM-T Easy Vector was ordered from Promega (Madison, WI, USA). Trans5α and BL21 (DE3) competent cells were acquired from TransGen Biotech (Beijing, China); Isopropyl b-D-1-thiogalactopyranoside (IPTG) and ampicillin were obtained from Sigma Chemical Co (St. Louis, MO); a-naphthol was ordered from Shanghai Chemical Reagent Company of Chinese Medical Group (Shanghai, China); a-naphthyl acetate (a-NA) was acquired from Shanghai Qingpu Synthetic Reagent Factory (Shanghai, China). Fast blue B salt was obtained from the Shanghai Equilibrated Reagent Factory (Shanghai, China); physostigmine was ordered from Fluka (Buchs, Switzerland); Coomassie blue G-250 was acquired from Amresco Co. (Solon, USA); bovine serum albumin (BSA) was obtained from Shanghai BioLife Science & Technology Co. (Shanghai, China); sodium dodecyl sulfate (SDS) was ordered from Sigma (Saint Louis, Missouri, USA); hydroxymethylaminomethane (Tris) was obtained from Shanghai Dingguo Biotech Development Co. (Shanghai, China). Triton X-100 was obtained from Amresco Co. (Solon, USA). Other chemicals and reagents were high quality commercially available products supplied by local suppliers.

### Total RNA isolation, synthesis of cDNA and dsRNA

Total RNA was extracted using RNeasy ® plus Micro Kit (Tiangen, Beijing, China) from 200 female adults (3–5d old) of *T. cinnabarinus* SS strain. To check the RNA quantity, the absorbance at 260 nm and the absorbance ratio of OD_260/280_ were measured with a Nanovue UV-Vis spectrophotometer (GE Healthcare, Fairfield, CT). The RNA quality was further confirmed by 1% agarose gel electrophoresis. The reverse transcription was carried out using PrimeScript ^®^ 1st Strand cDNA Synthesis Kit (Takara Biotechnology Dalian Co., Ltd., Dalian, China) and the synthesized cDNAs were stored at −20 °C. The available nucleotide sequences of the *T. cinnabarinus TCE2* gene (Genbank accession No. EU130462) were retrieved from the NCBI GenBank database. The forward primer and the reverse primer were designed to amplify cDNA fragments of *TCE2* (313 bp), which contained the T7 promoter region in both sense and antisense strands. The recombinant plasmids were used as a template with a protocol including preheating 94 °C for 3 min, followed by 35 cycles of 94 °C for 30 s, 60 °C for 30 s and 72 °C for 30 s, and a final extension step of 72 °C for 10 min. The sequence was confirmed by DNA sequencing (Beijing Genomic Institute, China). The Green Fluorescent Protein (GFP) (ACY56286) gene was used as a negative control. The primers GFPF and GFPR were used to amplify the GFP cDNA fragment. The primers used to synthesize dsRNA are listed in Supplementary Material: Table S1. All of the reagents and enzymes used for the dsRNA synthesis were from the TranscriptAid T7 High Yield Transcription Kit (Thermo scientific, Lithuania, EU). The dsRNA was further purified using the GeneJET RNA Purification Kit (Thermo scientific, Lithuania, EU). The final dsRNA was dissolved in nuclease-free water. The size of the dsRNA products was analyzed with 1% agarose gel electrophoresis, and quantified using a spectrophotometer and stored at −70 °C.

### Rearing on leaf-discs and dsRNA feeding

**Figure 7:**
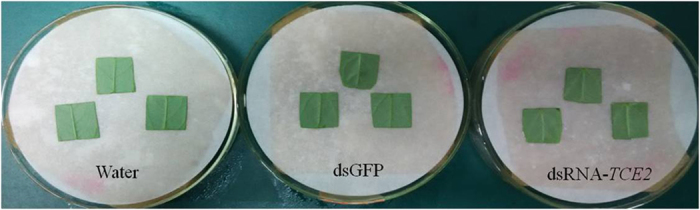
A picture of leaf-disc mediated dsRNA feeding.

Leaf-discs containing dsRNA-*TCE2* to knock down the expression of *T. cinnabarinus TCE2* gene were prepared as follows: cowpea leaves were cut to a 1.5 cm diameter feeding arena, incubated at 60 °C in lab oven for 3 minutes for dehydration, and then separately treated with water, dsGFP, or dsRNA-*TCE2* (1000 ng/μL) for 5 hours. After fully absorbed, the leaves were put on wet filter paper (Fig. 7). Thirty female adults (3–5d old and starved for 24 hours) were placed in each leaf-disc. The mites were reared under controlled growth conditions: 26 ± 1 °C, 35%–55% (RH), and 14:10 (L:D) photoperiod. After feeding for 48 hours, the mites were collected for the subsequent experiments.

### Quantitative real-time PCR (qPCR)

To verify the effectiveness of RNAi, approximately 200 female adults of SS, AbR, FeR, and CyR strains, after feeding with dsRNA for 48 hours, were collected for each sample, and were replicated for three times. The primers of qPCR for *TCE2* gene were designed by using primer 3.0 (http://frodo.wi.mit.edu/)^55^. *RPS18* (FJ608659) and *α-TUB* (FJ526336) were used as stable reference genes (Supplementary Material: Table S1)^56^. The qPCR was performed on an Mx3000P thermal cycler (Agilent Technologies, Inc., Wilmington, NC, USA) with 20 μL reaction mixtures containing 1 μL cDNA, 10 μL iQ™ SYBR® Green Supermix, 1 μL of each gene-specific primer (0.2 mM) and 7 μL ddH_2_O. The optimized qPCR protocol used for amplification was: 95 °C for 2 min, then 40 cycles of denaturation at 95 °C for 15 s, 60 °C for 30 s and elongation at 72 °C for 30 s. Finally, melt curve analyses (from 60 to 95 °C) were included at the end to ensure the consistency of the amplified products. The quantification of expression level was analyzed using the 2^−ΔΔCt^ method^57^. Differences in expression levels were analyzed by independent-sample *t*-test with a *p*-value < 0.05 in SPSS 19.0 (SPSS Inc., Chicago, USA).

### Susceptibility test of *T. cinnabarinus* to acaricides after RNAi of *TCE2*

The toxicities of three acaricides in the SS, AbR, FeR, and CyR strains were determined by the modified residual coated vial (RCV) method recommended by Van Leeuwen *et al.*^34^. The detailed bioassay procedure was described by Feng *et al.*^40^. Abamectin, fenpropathrin, and cyflumetofen were dissolved in acetone to concentrations of LC_30_ and LC_50_ (The LC_30_ and LC_50_ values of abamectin were 0.3 mg/L and 0.5 mg/L for SS, 2.0 mg/L and 3.3 mg/L for the AbR strain. The LC_30_ and LC_50_ values of fenpropathrin were 280.6 mg/L and 607.0 mg/L for SS, 27700.0 mg/L and 61580.0 mg/L for FeR strain. The LC_30_ and LC_50_ values of cyflumetofen were 1.5 mg/L and 2.5 mg/L for SS, 17.0 mg/L and 24.0 mg/L for CyR strain, respectively). The LC_30_ and LC_50_ values of three acaricides were used as the diagnostic dose for comparing the changes of the susceptibility to acaricides in *T. cinnabarinus* at 48 h post-feeding of dsRNA-*TCE2*, respectively. Thirty treated female mites were then transferred into the acaricide-coated centrifuge tube; each dose was performed in three replicates, including acetone as control. The mites were checked under anatomical microscope after 24 h rearing at 26 ± 1 °C, 35%–55% (RH), and 14:10 (L:D) photoperiod. Mites showing immobility or with legs irregularly trembling were considered dead. The statistical significance of mortality rate was calculated by independent-sample *t*-test with a *p*-value < 0.05 in SPSS 19.0 (SPSS Inc., Chicago, USA).

### Expression of *TCE2* gene

The specific primers of *TCE2* gene were designed (Supplementary Material: Table S1), which contained an N-terminal truncation (lacking the signal peptide sequence). The reverse transcribed cDNA was used as a PCR template to amplify *TCE2* coding sequence. PCR was performed with one cycle at 94 °C for 3 min, then 35 cycles at 94 °C for 30 s, 60 °C for 2 min and 72 °C for 2 min, followed by final step at 72 °C for 10 min. EcoR I and Xba I restriction enzyme cutting sites were incorporated into forward and reverse primers, which were used to sub-clone the coding sequence into an expression plasmid. The *TCE2* gene clone, ligated to the pGEM-T Easy Vector, was digested with EcoR I and Xba I, ligated into the pCold II expression vector linearized with the same enzymes, and transformed into Trans5α competent cells. After sequencing the positive clones to ensure in frame insertion, the pCold II-*TCE2* construct was transformed into *E. coli* (DE3) strain for protein expression and single colonies were inoculated into LB-ampicillin media. After overnight growth with shaking at 37 °C, cells were diluted 1:100 with LB-ampicillin media containing 100 mg L^−1^ ampicillin. Cells were shaken at 37 °C for 3 h until the OD600 reached 0.6–0.8 and cooled to room temperature. Then, IPTG was added into the culture broth to a final concentration of 0.1 mM. The culture was subsequently incubated at 15 °C for another 24 h at 180 rpm, and collected by centrifugation.

### Purification of TCE2 protein

Cells were collected by centrifugation at 4000 g for 20 min at 4 °C and resuspended in sterilized ice-cold buffer A (0.04 M phosphate buffer solution (PBS), pH 7.4, containing 0.5 M NaCl). The cells were disrupted by sonication (8 s, 150 W) on ice for 30 min and centrifuged at 10000 g for 15 min at 4 °C. The supernatant was applied to a Ni^2+^-NTA agarose gel column (Tiangen Biotech Co., Ltd., Beijing, China) for purification with a linear imidazole gradient of 40–500 mM in buffer A. The purified protein was detected by SDS-PAGE using a 5% (v/v) stacking gel and a 10% (v/v) resolving gel.

### Assay of enzymatic activity

Protein concentration was determined by Bradford’s method using bovine serum albumin as the standard^58^. The standard curve was firstly set using bovine serum albumin (BSA) as abscissa, and OD value as ordinate. The 50 μL working enzyme solution was mixed with Coomassie blue, and the control was PBS (0.04 M, pH 7.4), followed by an incubation at 37 °C for 10 min and measuring OD value at 595 nm in 96-well plates (Guangzhou JET Bio-Filtration Products Co., Ltd., Guangzhou, China) using a microplate reader (EON, BioTek Instruments Inc., Winooski, Vermont, USA). The OD value was converted to protein concentration based on the standard curve. The method reported by Van Asperen^59^ was adopted for testing recombinant TCE2 activities and endogenous esterase activities in *T. cinnabarinus*. For endogenous esterase activities measurement, 200 female adult mites were homogenized in 1.5 mL PBS (0.04 M, pH 7.4) on ice, then centrifugation at 10000 g for 10 min at 4 °C. The supernatant was placed on ice for testing. Using α-NA with physostigmine as substrate, this mixture was shaken for 10 s in the multiplate reader at 30 °C. After incubation for 10 min at 30 °C, the color developing agent (mixed as follows: mass fraction 5% SDS: mass fraction 1% fast blue B salt = 5:2 (v/v)) was added and the OD value was immediately recorded by measuring the optical density at 600 nm. The specific activities of recombinant TCE2 and esterases were calculated based on α-naphthol standard curve and protein concentration of enzyme source. The experiments were repeated for three times.

### Inhibition of acaricides

Substrate competitions of recombinant TCE2 between α-NA and three acaricides were valuated in an enzymatic assay. IC_50_ determination was also carried out in 96-well plates using a microplate reader. Assays were performed in a final volume of 200 μl consisting of 0.04 M PBS, pH 7.4, 0.1 mg/mL TCE2 protein, substrate concentration equal to the *Km* of the recombinant TCE2, and variable concentrations of tested acaricides. Different acaricide concentrations were prepared in acetone + 0.04 M PBS, pH 7.4 (5 + 95 by volume) containing 1% Triton X-100. This mixture was shaken for 10 s in the microplate reader at 30 °C, after incubation for 10 min at 30 °C, the color developing agent was added and the OD value was immediately recorded by measuring the optical density at 600 nm. For each combination of substrate and inhibitor at least three replicates were performed. IC_50_ values were calculated using SPSS 19.0 (SPSS Inc., Chicago, USA). Dixon plot analysis were performed at three different concentrations of α-NA (30, 90 and 150 μM), using four different concentrations of abamectin (0, 12, 115, 230 μM), fenpropathrin (0, 29, 287, 574 μM) and cyflumetofen (0, 23, 224, 447 μM) in order to determine the type of inhibition. The experiments were repeated for three times and results were analyzed with GraphPad prism 5 (GraphPad Software, Inc., USA).

### Hydrolysis activity of TCE2 protein

The ability of TCE2 to metabolize abamectin was determined by HPLC. Tested reactions were performed in a final volume of 1 ml consisting of 850 μL 5% acetonitrile, 100 μL 0.1 mg/mL TCE2 and 50 μL of 1000 mg/L abamectin. In the controlled reaction, 100 μL 0.04 M PBS (pH 7.4) was used to replace TCE2. Reactions were incubated at 30 °C, 250 rpm for 2 h. 750 μL reaction solution was transferred into 750 μL acetonitrile. Then, 5 μL of the supernatant was injected to glass HPLC vials at a flow rate of 1.2 mL/min at 30 °C. Abamectin was separated on a SunFire C18 (5 μm, 150 mm × 4.6 mm) reverse phase analytical column (Waters alliance 2695–2996). Reactions were run with an isocratic program 75% A: 25% B (A: 0.1% acetic acid in acetonitrile, B: 0.1% acetic acid in water) for 14 min. Abamectin elution was monitored by changes in absorbance at 245 nm, and was quantified by peak integration.

The ability of TCE2 to metabolize fenpropathrin was determined by GC. Tested reactions were performed in a volume of 1 ml (900 μL of 0.04 M PBS, pH 7.4; 100 μL of 0.1 mg/mL TCE2) and were added to 20 μl of 100 mg/L fenpropathrin in a centrifuge tube. Control reactions contained 1 ml of 0.04 M PBS, pH 7.4. Reactions were incubated at 30 °C, 250 rpm for 2 h; and then 2 mL of hexane was added with mixing. The mixture was allowed to settle for 30 min; and then the upper organic layer was transferred to another glass tube and brought up to 2 mL with hexane. An aliquot of the organic phase (1 mL) was analyzed for determining fenpropathrin content on an Agilent 6890 gas chromatography unit with an HP-5 capillary column (0.25 mm × 0.32 mm × 30.0 cm) and a micro-ECD detector (Agilent, USA) operated at 300 °C. Injections were performed in splitless mode and an inlet temperature of 250 °C. The carrier gas was nitrogen, at a flow rate of 50 ml/min. The GC oven was held at 80 °C for 30 s, then ramped up at 7 °C/min to 120 °C and then at 10 °C/min to 280 °C, where it was maintained for 10 min.

The ability of recombinant TCE2 to metabolize cyflumetofen was determined by HPLC. Tested reactions were performed in a final volume of 1 ml consisting of 820 μL 5% acetonitrile, 100 μl (0.05 mg/mL, 0.1 mg/mL) TCE2 protein, and 80 μL of 100 mg/L cyflumetofen. In the control reaction, recombinant protein was replaced by 100 μL 0.04 M PBS, pH 7.4. Reactions were incubated at 30 °C, 250 rpm for 2 h. 750 μL reaction solution was transferred into 750 μL acetonitrile. Then, 4 μL of the supernatant was injected at a flow rate of 0.25 mL/min at 35 °C. Cyflumetofen was separated on an Agilent SB-C18 (50 mm × 2.1 mm, 1.8 μm) reverse phase analytical column (Agilent, USA). Reactions were run with an isocratic program 55% A: 45% B (A: 0.2% ammonium acetate in acetonitrile, B: 0.2% formic acid in water) for 14 min. The degradation-rates of three acaricides under the role of the recombinant TCE2 were calculated from tested reaction and control reaction.

## Additional Information

**How to cite this article**: Shi, L. *et al.* Functional Analysis of Esterase *TCE2* Gene from *Tetranychus cinnabarinus* (Boisduval) involved in Acaricide Resistance. *Sci. Rep.* **6**, 18646; doi: 10.1038/srep18646 (2016).

## Supplementary Material

Supplementary table

## Figures and Tables

**Table 1:** The specific activity of recombinant TCE2 and esterases in mites.

Samples	Specific activity^a^ (nmol/mg pro.min^−1^) (α-NA)	K_m_(μM)	V_max_ (nmol/mg pro.min^−1^)
*T. cinnabarinus* (SS)	233.8 ± 20.3	–	–
Recombinant TCE2	483.3 ± 71.8	30.5 ± 5.9	448.2 ± 75.2
*T. urticae* (SS)	235.4 ± 10.3	–	–

^a^The specific activity of esterases in *T. urticae* was referred from Riga *et al.* (2014)^29^.

**Table 2:** Ability of acaricides to inhibit α-NA degradation catalyzed by TCE2.

Acaricides	Chemical type	IC_50_(μM)
Abamectin	Macrolide	54.16 ± 8.95
Fenpropathrin	Pyrethroid	235.15 ± 15.26
Cyflumetofen	Benzoyl acetonitrile	128.94 ± 12.67

**Table 3:** *In vitro* metabolism of acaricides by recombinant TCE2 protein.

Acaricides	*TCE2* protein (μg)	Metabolising rate (%)^a^
Abamectin	10.0	ND
Fenpropathrin	10.0	21.31 ± 2.29
Cyflumetofen	5.0	30.23 ± 5.82
	10.0	49.70 ± 11.45

^a^ND: not detected under assay conditions.
