# Supplementary material for: Functional Analysis of Esterase TCE2 Gene from Tetranychus cinnabarinus (Boisduval) involved in Acaricide Resistance
Source: Sci Rep. 2016 Jan 4;6:18646. doi: 10.1038/srep18646 (PMC4698665; doi:10.1038/srep18646)
Supplement: Supplementary table [file srep18646-s1.pdf]

Supplementary Information

Address Correspondence to:

Dr. Lin He
Key Laboratory of Entomology and
Pest Control Engineering
College of Plant Protection
Southwest University
Chongqing, China
Phone: +86-23-68251541
Fax: +86-23-68251514
E-mail: helinok@vip.tom.com

Dr. Qiang Xu
Department of Biology
ACU Box 27868
College of Arts and Sciences
Abilene Christian University
Abilene, Texas 79699 U.S.A.
Phone: +1-325-674-4883
Fax: +1-325-674-2009
E-mail: qxx07a@acu.edu

Functional Analysis of Esterase *TCE2* Gene from *Tetranychus cinnabarinus* (Boisduval) involved in Acaricide Resistance

Li Shi[1*], Peng Wei[1*], Xiangzun Wang[1*], Guangmao Shen[1], Jiao Zhang[1], Wei Xiao[1], Zhifeng Xu[1], Qiang Xu[2¶], Lin He[1¶]

[1]Key Laboratory of Entomology and Pest Control Engineering, College of Plant Protection, Southwest University, Chongqing, China;

[2]Department of Biology, Abilene Christian University, Abilene, Texas, U.S.A.

*These three authors contributed equally to this work.

¶ To whom correspondence should be addressed

Supplementary Information

Table S1. Primers used for RNAi, heterologous expression and qPCR

Genes	GenBank No.	Experiments	Primer names and sequences (5' to 3')	Product length (bp)	Amplification efficiency (%)
GFP	ACY56286	RNAi	F:taatacgactcactatagggGCAGTTCTTGTTGAATTAGATG<br>R:taatacgactcactatagggTTTGGTTTGTCTCCCATGATG	476	-
TCE2	EU130462	RNAi	F:taatacgactcactatagggTCGGCGGCAATCCTGATCAA<br>R:taatacgactcactatagggTCACAGGGTAAAATTCAGCA	313	-
		Heterologous expression	F: CCGGAATTCGATGTAAGTGAGGAACCAAC<br>R: CCGCTCGAGTCAAAGTTCATCATGGT	1614	-
		qPCR	F: CCATCCAAAACACCGATTTC<br>R: CTTCCCCAAGCATTCATCAT	161	91.3
RPS18	FJ608659	qPCR	F: ACGTGCTGGTGAACTTACCGAAGA<br>R: TGCCTATTCAAGAACCAAAGTGGG	99	98.5
α-TUB	FJ526336	qPCR	F:ACTACGCTCGTGGCCACTATACAA<br>R:ACCAGATCCAGTTCCACCTCCAAA	140	97.4

Underlines indicate EcoR I and Xba I restriction enzyme cutting sites
